# Physician perspectives and compliance with patient advance directives: the role external factors play on physician decision making

**DOI:** 10.1186/1472-6939-13-31

**Published:** 2012-11-21

**Authors:** Christopher M Burkle, Paul S Mueller, Keith M Swetz, C Christopher Hook, Mark T Keegan

**Affiliations:** 1 Department of Anesthesiology, Mayo Clinic, 200 First Street SW, Rochester, MN 55905, USA; 2 Division of General Internal Medicine, Department of Medicine, Mayo Clinic, Rochester, MN, USA; 3 Division of Hematology, Department of Medicine, Mayo Clinic, Rochester, MN, USA

**Keywords:** Advance directive, Physician decision making, Patient preference

## Abstract

**Background:**

Following passage of the Patient Self Determination Act in 1990, health care institutions that receive Medicare and Medicaid funding are required to inform patients of their right to make their health care preferences known through execution of a living will and/or to appoint a surrogate-decision maker. We evaluated the impact of external factors and perceived patient preferences on physicians’ decisions to honor or forgo previously established advance directives (ADs). In addition, physician views regarding legal risk, patients’ ability to comprehend complexities involved with their care, and impact of medical costs related to end-of-life care decisions were explored.

**Methods:**

Attendees of two Mayo Clinic continuing medical education courses were surveyed. Three scenarios based in part on previously court-litigated matters assessed impact of external factors and perceived patient preferences on physician compliance with patient-articulated wishes regarding resuscitation. General questions measured respondents’ perception of legal risk, concerns over patient knowledge of idiosyncrasies involved with their care, and impact medical costs may have on compliance with patient preferences. Responses indicating strength of agreement or disagreement with statements were treated as ordinal data and analyzed using the Cochran Armitage trend test.

**Results:**

Three hundred eighty-eight of 951 surveys were completed (41% response rate). Eighty percent reported they were likely to honor a patient’s AD despite its 5 year age. Fewer than half (41%) would honor the AD of a patient in ventricular fibrillation who had expressed a desire to “pass away in peace.” Few (17%) would forgo an AD following a family’s request for continued resuscitative treatment. A majority (52%) considered risk of liability to be lower when maintaining someone alive against their wishes than mistakenly failing to provide resuscitative efforts. A large percentage (74%) disagreed that patients could not appreciate complexities surrounding their care while 69% agreed that costs should never impact a physician’s decision as to whether to comply with a patient’s AD.

**Conclusions:**

Our findings highlight the impact, albeit small, external factors have on physician AD compliance. Most respondents based their decision on the clinical situation at hand and interpretation of the patient’s initial wishes and preferences expressed by the AD.

## Background

Prior to the 1970s, medical care of the seriously ill was generally physician-directed by the ethical principles of beneficence and non-malfeasance [1]. Physicians (and other care providers) often provided treatments without first assessing patients’ values or goals of care. This approach changed substantially following the highly publicized 1976 New Jersey Supreme Court decision of *in re Quinlan*, which held that a patient or his or her guardian had the right to refuse unwanted life-sustaining treatment even if doing so resulted in death. Public response to this case and subsequent cases [2-4] gave rise to the notion that patients ought to have a means of communicating their health care related values and goals in the event they are unable to communicate for themselves [5]. Indeed, following the Supreme Court’s *Cruzan* decision, the Patient Self Determination Act (PSDA) was passed in 1990, requiring health care institutions that receive Medicare and Medicaid funding to inform patients of their right to make their health care preferences known through execution of a living will and/or to appoint a surrogate-decision maker [5,6]. All U.S. states and the District of Columbia have laws that recognize advance directives (ADs) [5].

Despite the transition from paternalistic decision making on the part of health care providers to decision making more centered on patient autonomy, the advent of ADs as a means of consistently and accurately reflecting patient wishes has been less successful [5-7]. Health care providers have struggled to align the importance of patient autonomy with treatments based on beneficial care [5,8-11]. When considering whether to provide life-supporting measures, physicians may be influenced by patient preferences, medical considerations and external factors [10-12]. External factors may include family wishes, financial considerations, physician characteristics and fear of legal liability [7-11,13-17]. Described as “interests of people other than the patient”, these matters can lead to ethical conflicts involving patient care [7,10].

In this study, we evaluated the impact of these external factors and perceived patient preferences on physicians’ decisions to honor or forgo previously established ADs. In addition, we explored physicians’ views regarding legal risk, concerns over patients’ ability to comprehend complexities involved with their care, and the impact of medical costs related to end-of-life care decisions.

## Methods

### Setting and participants

This study was approved by Mayo Clinic’s Institutional Review Board. Each year, Mayo School of Continuing Professional Development conducts several comprehensive internal medicine continuing education courses. Attendees of two of these courses (Mayo Internal Medicine Board Review and Mayo Clinical Reviews), which were conducted in Rochester MN, were surveyed using a Scantron® survey based system. The survey instrument is in Additional file 1.

Three scenarios based, in part, on previously court-litigated matters were employed to assess the impact of external factors and perceived patient preferences on physician compliance with patient-articulated wishes regarding resuscitation (Additional file 1). Scenario 1 presented respondents with an AD that had been signed in the past as the patient prepared for elective surgery and who, years later, presented with an acute unrelated problem.

"Scenario 1: A 62 year old patient with a history of hypertension and type 2 diabetes presents to the Emergency Department complaining of “not feeling well”. His initial blood pressure reading by cuff is 240/110. Intravenous blood pressure therapy is provided by the Emergency Room staff after which he is transferred to a monitored hospital bed for close observation. Shortly following admission, the patient complains of a severe headache. Your work-up confirms that the patient has suffered a massive cerebral vascular accident (CVA). He is now is in severe respiratory distress. A trial of non-invasive ventilation has failed and the patient now requires intubation. An advanced directive signed by the patient 5 years ago prior to knee surgery indicates that the patient is “Do not resuscitate” (DNR) / “Do not intubate” (DNI)."

Scenario 2 aimed to assess the impact that illness acuity had on physician decision-making in the setting of an AD that reflected patient wishes to “pass away in peace”.

"Scenario 2: A 65 year old patient with well controlled hypertension and who has signed an advance directive order stating a wish to “pass away in peace” arrives to the Emergency Department complaining of “chest pain”. He is admitted to the cardiac telemetry unit pending a full work-up for cardiac ischemia. Shortly following arrival to the unit, he falls into ventricular fibrillation. A “Code” is called and upon your arrival to the bedside, the patient is apneic and requiring mask ventilation by the nursing staff."

Scenario 3 explored the influence of family demands that conflicted with a patient’s AD had on decision making.

"Scenario 3: A 68 year old patient with hypertension, diabetes, end stage renal disease on dialysis and acute lymphocytic leukemia arrives to the Emergency Department febrile, tachypneic and hypotensive. He is transferred to the intensive care unit (ICU) you are covering and upon arrival becomes asystolic. The hospital electronic charting system shows that the patient completed and signed an advance directive indicating “Do not resuscitate” (DNR) / “Do not intubate” (DNI) wishes during a recent visit with his oncologist. The wife of the patient has demanded that you “disregard the advance directive and do everything you can to save my husband”."

A complete description of each scenario can be found in Additional file 1.

General questions were used to measure respondents’ agreement with statements pertaining to perceptions of legal risk, concerns over patient knowledge of the idiosyncrasies involved with their care, and the impact that medical costs may have on compliance with patient preferences. A pilot study involving Mayo Clinic Department of Medicine staff physicians was initially undertaken to analyze the performance of both scenario-based and general question set design.

### Statistical analysis

Responses which indicated “prefer not to answer” were treated as missing data. In addition to analysis using descriptive statistics, the responses indicating the strength of agreement or disagreement with statements were treated as ordinal data and analyzed using the Cochran Armitage trend test. Responses to questions 5–19 were also grouped into two levels, depending on the question: Very/somewhat likely and very/somewhat unlikely; Very/somewhat important and very/somewhat unimportant; Strongly agree/agree and Strongly disagree/disagree. These binary groupings were treated as categorical data for Chi-square and Fisher exact test analyses, as appropriate, depending on the numbers in each cell. A *P-*value of less than 0.05 was considered significant.

## Results

Overall, 388 surveys were completed by 951 attendees (response rate, 41%). The demographic characteristics (gender, age, type of service, years in practice) of respondents appear in Table 1. For the purpose of discussion of the results, Somewhat likely/Very likely and Somewhat unlikely/Very unlikely are reported as Likely and Unlikely, respectively. Similarly, we report Important and Unimportant, and Agree and Disagree.

**Table 1:** Demographics of Respondents

**Gender**	
Female	147 (38%)
Male	240 (62%)
“Prefer not to answer” or left blank	1 (<1%)
**Age (years)**	
21-35	53 (14%)
36-50	97 (25%)
51-65	174 (45%)
> 65	61 (16%)
“Prefer not to answer” or left blank	3 (<1%)
**Specialty focus**	
Primary care (general focus)	286 (74%)
Primary care (subspecialty focus)	36 (9%)
Intensive care	8 (2%)
Other	55 (14%)
“Prefer not to answer” or left blank	3 (<1%)
**Practice duration (years)**	
0-5	69 (18%)
6-15	75 (20%)
16-30	133 (35%)
> 30	105 (27%)
“Prefer not to answer” or left blank	6 (<1%)

### Scenario 1 (Old AD, used subsequently)

A majority (80%) of respondents reported they were likely to honor the patient’s AD and not intubate the patient suffering from a massive stroke. (Table 2) Sixty percent of all respondents considered the age of the AD to be important in their decision making. This figure was even higher (69%) in those who were unlikely to adhere to the AD. (Table 3) About one-third (37%) of all surveyed reported fear of legal liability as a factor in their decision *(P* = 0.05). However, this figure did not vary among those who reported they would or would not comply with the patient’s AD *(P* = 0.21). Physician age and number of years in practice did not influence answers provided to questions in Scenario 1, although older physicians were more likely to have a measured view of the importance of legal considerations with fewer responding “very important” or “very unimportant” to the consideration of legal liability *(P =* 0.03) (Table 4).

**Table 2:** Scenario responses including impact of advance directive characteristics on final decision making

**Scenario 1**		
**Honor Advance Directive**		
Likely	312 (80%)	
Unlikely	48 (12%)	
Unsure	28 (7%)	P < 0.01
**Signing 5 years ago, prior to elective surgery**		
Important	232 (60%)	
Unimportant	77 (20%)	
Unsure	79 (20%)	P <0.001
**Fear of legal liability**		
Important	145 (37%)	
Unimportant	136 (35%)	
Neither	107 (28%)	P = 0.05
**Scenario 2**		
**Honor Advance Directive**		
Likely	160 (41%)	
Unlikely	175 (45%)	
Unsure	53 (14%)	P < 0.001
**Acuity of illness does not mirror “pass away in peace”**		
Important	280 (72%)	
Unimportant	50 (13%)	
Neither	58 (15%)	P < 0.001
**Fear of legal liability**		
Important	159 (41%)	
Unimportant	100 (26%)	
Neither	129 (33%)	P < 0.001
**Scenario 3**		
**Honor Advance Directive**		
Likely	288 (74%)	
Unlikely	67 (17%)	
Unsure	33 (8%)	P < 0.001
**Spouse’s Demand**		
Important	213 (55%)	
Unimportant	106 (27%)	
Neither	69 (18%)	P < 0.001
**Fear of legal liability**		
Important	206 (53%)	
Unimportant	89 (23%)	
Neither	93 (24%)	P < 0.001

Consistent with the Results section, we report Somewhat likely/Very likely and Somewhat unlikely/Very unlikely as Likely and Unlikely, respectively. Similarly, we report Important and Unimportant.

P-values reflect comparisons between the 3 responses.

**Table 3:** Influence of a variety of factors on the likelihood of compliance with advance directive

**Scenario**	**Question**	**Honor AD?**			**P-value**
		**Likely**	**Unlikely**	**Unsure**	
**1**	**Signing 5 years ago prior to elective surgery**				< 0.046
	Important	176 (56%)	33 (69%)	23 (82%)	
	Unimportant	68 (22%)	7 (15%)	3 (11%)	
	Neither	68 (22%)	7 (15%)	2 (7%)	
	**Fear of legal liability**				0.207
	Important	111 (36%)	25 (52%)	32% (9)	
	Unimportant	115 (37%)	11 (23%)	36% (10)	
	Neither	86 (28%)	12 (25%)	9 (32%)	
**2**	**Acuity of illness does not mirror “pass away in peace”**				<0.01
	Important	90 (56%)	151 (86%)	39 (74%)	
	Unimportant	35 (22%)	12 (7%)	3 (6%)	
	Neither	35 (22%)	12 (7%)	11 (21%)	
	**Fear of legal liability**				0.085
	Important	74 (46%)	62 (35%)	23 (43%)	
	Unimportant	39 (24%)	53 (30%)	8 (15%)	
	Neither	47 (29%)	60 (34%)	22 (42%)	
**3**	**Spouse’s demand**				< 0.01
	Important	141 (49%)	52 (77%)	20 (61%)	
	Unimportant	92 (32%)	11 (16%)	3 (9%)	
	Neither	55 (19%)	4 (6%)	10 (30%)	
	**Fear of legal liability**				< 0.006
	Important	144 (50%)	42 (63%)	20 (61%)	
	Unimportant	71 (25%)	17 (25%)	1 (3%)	
	Neither	73 (25%)	8 (12%)	12 (36%)	

Consistent with the Results section, we report Somewhat likely/Very likely and Somewhat unlikely/Very unlikely as Likely and Unlikely, respectively. Similarly, we report Important and Unimportant.

Cells are Number (% of column). For example, of the 312 respondents who were Likely (i.e. Very Likely or Somewhat Likely) to honor the advance directive in Scenario 1, 176 (56% of the 312) considered the fact that the advance directive was signed 5 years before to be Important (i.e. Important or Very Important).

P-values reflect Chi-square or Fisher exact test comparisons.

**Table 4:** Impact of respondent age and years in practice on the likelihood of compliance with advance directive

**Scenario 1**	**Age (years)**			**Years in Practice**		
	**21-50**	**> 50**	**P-value**	**0-15**	**>15**	**P-value**
**Honor AD**			0.841			0.586
Likely	123 (82%)	187 (80%)		115 (80%)	193 (81%)	
Unlikely	17 (11%)	30 (13%)		18 (13%)	28 (12%)	
Unsure	10 (7%)	18 (8%)		11 (8%)	17 (7%)	
**Signing 5 years ago prior to elective surgery**			0.239			0.608
Important	84 (56%)	146 (62%)		87 (60%)	140 (59%)	
Unimportant	36 (24%)	40 (17%)		31 (22%)	46 (19%)	
Neither	30 (20%)	49 (21%)		26 (18%)	52 (22%)	
**Fear of legal liability**			0.114			0.083
Important	65 (43%)	79 (34%)		60 (42%)	84 (35%)	
Unimportant	51 (34%)	85 (36%)		53 (37%)	82 (34%)	
Neither	34 (23%)	71 (30%)		31 (22%)	72 (30%)	
**Scenario 2**	**Age (years)**			**Years in Practice**		
	**21-50**	**> 50**	**P-value**	**0-15**	**>15**	**P-value**
**Honor AD**			0.181			0.279
Likely	67 (45%)	93 (40%)		61 (42%)	97 (41%)	
Unlikely	59 (39%)	114 (49%)		57 (40%)	115 (48%)	
Unsure	24 (16%)	28 (12%)		26 (18%)	26 (11%)	
**Acuity of illness does not mirror “pass away in peace”**			< 0.033			0.056
Important	102 (68%)	176 (75%)		101 (70%)	177 (74%)	
Unimportant	17 (11%)	33 (14%)		15 (10%)	33 (14%)	
Neither	31 (21%)	26 (15%)		28 (19%)	28 (12%)	
**Fear of legal liability**			0.258			0.084
Important	69 (46%)	89 (38%)		70 (49%)	86 (36%)	
Unimportant	34 (23%)	66 (28%)		31 (22%)	69 (29%)	
Neither	47 (31%)	80 (34%)		43 (29%)	83 (35%)	
**Scenario 3**	**Age (years)**			**Years in Practice**		
	**21-50**	**> 50**	**P-value**	**0-15**	**>15**	**P-value**
**Honor AD**			0.834			0.117
Likely	110 (73%)	177 (75%)		108 (75%)	178 (75%)	
Unlikely	26 (17%)	40 (17%)		21 (15%)	43 (18%)	
Unsure	14 (9%)	18 (8%)		15 (10%)	17 (7%)	
**Spouse’s demand**			< 0.012			< 0.012
Important	74 (49%)	138 (59%)		66 (46%)	143 (60%)	
Unimportant	54 (36%)	52 (22%)		53 (37%)	53 (22%)	
Neither	22 (15%)	45 (19%)		25 (17%)	42 (18%)	
**Fear of legal liability**			0.864			0.694
Important	81 (54%)	125 (53%)		74 (51%)	128 (54%)	
Unimportant	36 (24%)	53 (23%)		36 (25%)	53 (22%)	
Neither	33 (22%)	57 (24%)		34 (24%)	57 (24%)	

Consistent with the Results section, we report Somewhat likely/Very likely and Somewhat unlikely/Very unlikely as Likely and Unlikely, respectively. Similarly, we report Important and Unimportant.

Cells are Number (% of column). For example, in Scenario 1, of the 150 respondents who were aged between 21 and 50, 123 (82% of the 150) were Likely (i.e. Very Likely or Somewhat Likely) to honor the advance directive.

P-values reflect Chi-square or Fisher exact test comparisons.

### Scenario 2 (AD expresses wish to die peacefully, acute situation encountered)

Fewer than half (41%) of respondents reported they would honor the AD of a patient in ventricular fibrillation who had expressed a desire to “pass away in peace.” (Table 2) A large majority (86%) considered it important that the patient’s request did not mirror the acuity of the immediate condition requiring intervention. The results for Question 8 differed by gender, with female physicians more likely than male physicians to express uncertainty as to how they would proceed based on the information provided. (*P* = 0.01) Respondents older than age 50 years were more likely to consider the acuity of the condition as important. (*P* = 0.03; Table 4).

The decision to defibrillate the patient or not was independent of the fear of legal liability (*P* = 0.07). (Tables 3 and 4) The fear of legal liability was not influenced by gender, age, practice type or duration of practice.

### Scenario 3 (Family in conflict with patient’s stated preferences)

In the hypothetical case of the critically ill patient with leukemia, three-fourths of respondents (74%) reported that they would honor the AD despite the spouse’s request for continued resuscitative care. (Table 2) Among those who would not adhere to the AD, 78% considered the spouse’s demand important to their decision. (Table 3) Respondents older than 50 years were more likely (59% versus 49%, *P* = 0.01) to be influenced by the wife’s request than respondents age 50 years and younger. (Table 4) Similarly, 60% of those physicians in practice for more than 15 years reported the spouse’s request as important to their decision compared with 46% of those with less experience (*P* = 0.01). (Table 4) Overall, fear of legal liability was important to more of the respondents (53%) than noted in either of the other two patient scenarios (*P* < 0.01). (Table 3) Although a higher percentage of respondents were influenced by a fear of liability amongst those who would not honor the AD (63%) compared with those who would (50%), the difference was not statistically significant (*P* = 0.42). (Table 3) The fear of legal liability was independent of age or years of practice (Table 4).

Two questions are common to each of the three scenarios. Questions 5, 8 and 11 concern the likelihood of honoring the advance directive. Questions 7, 10 and 13 ask about the legal fears of the decision. Of the 388 respondents, 312 stated that they were likely or very likely to honor the advance directive in Scenario 1. Of these, only 139 were also likely/very likely to honor the advance directive in Scenario 2, and only 108 were also likely/very likely to honor the advance directive in Scenario 3. Thus, 108 of the 388 respondents (28%) answered consistently across scenarios regarding their likelihood of honoring the advance directive. Although the fear of legal liability was very important or important to 37%, 41% and 53% of respondents for Scenarios 1, 2, and 3, respectively, only 39 respondents (10% of the total), consistently answered that the fear of legal liability was (very) important in each Scenario. There was similar scenario-dependency for (very) unimportant, with only 15 of 388 respondents (4%) minimizing legal concerns in all scenarios.

### General questions

Table 5 shows the results of the general (non-scenario-based) questions. The responses to this set of general questions were not impacted by demographic factors (gender, age, years in practice).

**Table 5:** Respondent level of agreement with general topics involving advance directives

**Statement Presented to Responding Physicians**	**Agree**	**Disagree**	**Neither**	**P-value**
Liability risk less for maintaining someone alive against their will than mistakenly allowing them to die.	200 (52%)	116 (30%)	72 (19%)	< 0.01
Comfort measures only should allow physicians to continue life support measures.	74 (19%)	262 (67%)	53 (14%)	< 0.01
“No life support” should be interpreted literally.	248 (64%)	87 (22%)	53 (14%)	< 0.01
Physicians should be allowed to provide care independent of the advance directive as patients do not have the knowledge to best appreciate the idiosyncrasies involved with the practice of medicine.	58 (15%)	287 (74%)	43 (11%)	< 0.01
Physicians should only be legally liable when they intentionally disregard a patient’s AD.	216 (56%)	84 (22%)	88 (23%)	< 0.01
The financial cost of providing medical care should never impact a decision to honor or forgo expressed wishes noted in an AD.	267 (69%)	65 (17%)	56 (14%)	< 0.01

Consistent with the Results section, we report Strongly Agree/Agree as Agree, and Strongly Disagree/Disagree as Disagree.

## Discussion

The results of our survey suggest that physician adherence to ADs is situation-specific, and that in rapidly reversible conditions (i.e. Scenario 2) physicians believe their judgment supersedes previously-specified patient instructions. The fear of legal action did not appear to be a major factor in the compliance or non-compliance with ADs, except in the case of Scenario 3 in which there was family disagreement with patient wishes.

Although ADs may not offer the results initially envisioned by those who advocated for their existence, they nonetheless may provide a valuable, societally-acceptable approach to communicating one’s desires regarding end-of-life care goals and preferences [5]. Similar to other reports [5,7-9,11,15], our study found that despite knowledge of their existence, physicians do not consistently honor patients’ ADs. In our survey, the temporal remoteness of the AD and the reversibility of the immediate condition appeared to hold greater relevance for clinicians’ decision making.

A majority of physicians surveyed would refuse to provide emergency care to a patient who completed an AD before undergoing a previous routine surgical procedure (Scenario 1) but would deliver treatment in a case where the AD reflected a patient’s wish to pass away in peace (Scenario 2). Like physicians and other health care providers, courts have been confronted with disputes over the appropriateness of providing emergency treatment to patients who have previously requested limits to their care through ADs [5]. One representative Ohio court judgment held that an earlier decision by a patient to forego life-saving measures could carry over to an emergency condition only after it was determined that the patient had both knowledge and understanding of how the two settings may differ [18]. By example, the court acknowledged the difference between a terminally ill patient who had previously requested to die in peace and now suffers injuries sustained in an automobile accident and the treatment of an emergency condition developing from their terminal condition. In line with this judicial reasoning, respondents we surveyed appeared to be concerned that the request to “pass away in peace” in Scenario 2 was effectively different from the condition that brought the patient to the hospital that day.

### Legal concerns influencing decisions on advance directives

Over half (52%) of the respondents in our study agreed that the risk of liability was lower when maintaining someone alive against their wishes than in mistakenly failing to provide resuscitative efforts. Prior reports also suggest that the risk of legal liability may influence a practitioner’s decision regarding end-of-life care and resuscitative efforts [15,19]. In a survey of emergency medicine physicians, 58% of respondents stated that their decisions regarding resuscitation were largely influenced by fears of litigation or criticism [15]. Although 80% admitted legal concerns should not influence physician decision-making regarding resuscitation, 92% reported that the present legal environment did influence their practice [15].

Although claims have been filed against hospitals and physicians alike for damages arising from delivery of care against the express wishes of patients, it should offer comfort to providers that courts have struggled when deciding if and how to award compensation for “continued living” [5,19]. In *Anderson v. St. Francis-St. George Hospital, Inc*, a patient, who had provided clear instructions upon admission to the emergency room to forgo extraordinary life-sustaining treatment, sued for damages resulting from a stroke sustained following cardiac defibrillation for ventricular fibrillation [5,19-21]. The *Anderson* court held that such damage awards were unavailable unless the patient could show that “defibrillation itself caused or contributed to [his] stroke in any way other than simply prolonging his life ” [5,21]. Instead, damages would only be awarded for direct injuries resulting from resuscitation efforts such as burns due to defibrillation.

When confronted with a spouse’s wishes that conflict with those provided by the patient’s AD, over half of respondents (53%) considered the threat of legal liability as important or very important to their decision. The fear of liability was more prevalent than expressed in the two prior patient scenarios (37% and 42%, respectively). Furthermore, a majority of those failing to honor the AD in Scenario 3 were strongly influenced by the spouse’s request for continued care (77%) and potential risk of future liability (63%). Our results remain in line with other reports suggesting that due to the limited number of cases filed against practitioners for failing to abide by a patient’s AD, along with the courts’ general unwillingness to award damages for continued life, practitioners often consider the risk of mistakenly failing to deliver treatment greater than providing care against the wishes of patient or surrogate [5].

With many confounding factors influencing physician decisions to honor or forego patient ADs, some have advocated that a better means of ensuring physician compliance with patient wishes is to limit legal liability to only those instances where intentional disregard for the instructions provided in the AD occurs [5]. Despite proposed benefits, only 56% of respondents agreed with this approach to balancing liability. The majority of respondents (74%) disagreed that the complexity of medical care should limit the level of patient participation in decisions at the end-of-life. Perhaps this strong view among those surveyed could help explain why a standard of liability limited to the *intentional* disregard for patient wishes alone did not gain greater support.

### Family influence on physician compliance with advance directives

Almost three-quarters of physicians surveyed (74%) in our study stated they would continue to honor an AD despite the spouse’s request to the contrary. However, among those who reported that they would not comply with the patient’s AD (17%), a large majority (77%) considered the spouse’s demand important or very important in their decision. Demands for care by the family that are at odds with those expressed by patient’s AD, have been considered the leading cause for physician noncompliance with ADs [6]. However, the general lack of spousal influence on those we surveyed may reflect problems others in the past have highlighted [1]. Family members and surrogates often fail to accurately predict a patient’s treatment wishes by overestimating the patient’s desire for continued treatment [22].

Older (over 50 years of age) physicians and those with more practice experience (>15 years) were more influenced by the spouse’s demand than their younger colleagues. Prior studies suggest that physician age may impact the level of influence a family’s appeal has on physician practices [13]. Hinkka *et al.* reported that while family requests had a greater initial influence on younger physicians’ care decisions, any age-specific trends tended to fade with the introduction of an AD [13]. As those we surveyed were aware that an AD existed, any bias on the part of younger physicians to respect family requests may have been nullified.

### Limitations of care

At our institution, and in many other institutions, there is a distinction between “comfort measures only” and a desire not to undergo cardiopulmonary resuscitation (i.e. to be “Do Not Resuscitate/Do Not Intubate” [DNR/DNI]). In the former, all treatment measures that are not intended to alleviate discomfort are discontinued, except for the use of opiates, benzodiazepines, anti-sialogogues and nursing cares are continued to maximize comfort. This practice is consistent with the thoughts of the 67% of respondents who disagreed or strongly disagreed with the statement that "comfort measures only should allow physicians to continue life support measures." (Table 5) In contrast, when a patient has made a decision to not be resuscitated or intubated (i.e. they are DNR/DNI), *but has****not****decided to be “comfort measures only”*, this can still be consistent with aggressive therapy directed towards a patient’s goals of care (i.e. chemotherapy, radiation, antibiotics, pressors, etc.) [23-27]. Withholding treatment at the end-of-life does not necessarily suggest that all care should be discontinued and patients who are DNR/DNI do not necessarily wish to forego ICU care. Several states have enacted laws ensuring end-of-life care and surrogate-decision making occurs in line with a patient’s expressed wishes to promote a patient’s dignity [22]. Practitioners should be aware that the blurring of lines between treatment and comfort measures not only stands at odds with patient autonomy and the right to decision making, but comes with some additional (while rare) legal risk [5]. For example, a Michigan court awarded damages to the family of a patient who awoke from a coma but remained in a vegetative state after a hospital provided care in excess of the patient’s health care proxy requests for comfort measures only [5].

### Costs of care

Two-thirds of those we surveyed (69%) agreed that costs should never impact a physician’s decision as to whether to comply with a patient’s AD. According to a recent report released by the Agency for Healthcare Research and Quality, 1% and 5% of the population accounted for 22% and 50% of health care expenditures respectively (using 2009 data) [28]. Forty-percent of health-care costs involved care delivered to patients 65 years of age or older. Our results appear in line with prior surveys of physicians finding that a majority object to the use of cost-effectiveness data to determine the benefit of treatment provided to patients [29]. Others have suggested that physicians may be unwilling to enter the controversial domain they believe better belongs to politicians, administrative bodies and health care advocates [29].

Our study has methodological limitations. Given that 41% of surveys were returned, our results may not be generalized to all clinicians who are commonly confronted with the issue of deciding on whether to follow or forego patient ADs. Another potential limitation of our study is that those surveyed were not asked if they are commonly confronted with issues related to patient advance directives during their normal course of daily practice, although in general, many of the general internists who attend these meetings are hospitalists and commonly address these issues.

## Conclusion

Clinicians may be faced with instances where providing care to patients is inconsistent with the desires expressed in an AD. This study set out to explore clinician compliance with patient AD’s and to determine whether particular external factors may influence decisions to either honor or forego pre-set directives. When confronted with a specific patient scenario, most of the provider decisions in our study were influenced by the entirety of the clinical situation (e.g. temporal remoteness in relation to the AD and the reversibility of the immediate condition) rather than a literal application of the AD. Furthermore, most clinician decisions were made without concern for legal liability or influenced by demands by families to continue care. Our findings confirm that a majority of clinicians appreciate that while a patient may wish to forego end-of-life treatment, this does not equate to sparing comfort care measures. Also, most clinicians we surveyed agreed that their treatment decisions should not be influenced by the monetary cost required in providing this care.

Until a more reliable system can be developed that enables a patient’s desires to be fully expressed under a multitude of unique clinical scenarios, physicians will be required to interpret, as they did in our study, the interaction between a patient’s immediate medical condition and their prior requests as documented through ADs.

## Competing interests

All authors: No financial or non-financial competing interests.

## Authors’ contributions

CB conceived of the study, participated in its design and coordination and helped to draft the manuscript. MK participated in the design of the study, performed the statistical analysis and helped draft the manuscript. PM participated in the design of the study, coordinated survey logistics and helped draft the manuscript. KS participated in design of the study and helped draft the manuscript. CH participated in the design of the study and helped coordinate survey logistics. All authors read and approved the final manuscript.

## Source of funding

All authors and manuscript preparation: Departmental and Institutional resources.

## Pre-publication history

The pre-publication history for this paper can be accessed here:

http://www.biomedcentral.com/1472-6939/13/31/prepub

## Supplementary Material

Additional file 1 **Physician Questionnaire.** Physician questionnaire including three patient oriented scenarios and general question set. Click here for file
