# Supplementary material for: Physician perspectives and compliance with patient advance directives: the role external factors play on physician decision making
Source: BMC Med Ethics. 2012 Nov 21;13:31. doi: 10.1186/1472-6939-13-31 (PMC3528447; doi:10.1186/1472-6939-13-31)
Supplement: Additional file 1 — Physician Questionnaire. Physician questionnaire including three patient oriented scenarios and general question set. [file 1472-6939-13-31-S1.pdf]

Advance Directive Survey IM

This is an anonymous survey and your responses will remain confidential. Please <u>fill in circles completely with a black ink pen or # 2 pencil.</u>

	Female		Male		Prefer not to Answer
1. **What is your gender?**	◯		◯		◯

	21- 35	36 - 50	51 - 65	>65	Prefer not to Answer
2. **What is your age?**	◯	◯	◯	◯	◯

	Primary Care (primary/general focus)	Primary Care (subspecialty focus)	Intensive Care	Other	Prefer not to Answer
3. **What is your area of primary practice?**	◯	◯	◯	◯	◯

	0-5	6-15	16-30	>30	Prefer not to Answer
4. **How many years have you been in practice?**	◯	◯	◯	◯	◯

PLEASE ANSWER THE QUESTIONS BELOW RELATED TO THE FOLLOWING PATIENT SCENARIOS (1 - 3):

<u>SCENARIO 1:</u> A 62 year old patient with a history of hypertension and type 2 diabetes presents to the Emergency Department complaining of "not feeling well". His initial blood pressure reading by cuff is 240/110. Intravenous blood pressure therapy is provided by the Emergency Room staff after which he is transferred to a monitored hospital bed for close observation. Shortly following admission, the patient complains of a severe headache. Your work-up confirms that the patient has suffered a massive cerebral vascular accident (CVA). He is now in severe respiratory distress. A trial of non-invasive ventilation has failed and the patient now requires intubation. An advanced directive signed by the patient 5 years ago prior to knee surgery indicates that the patient is "Do not resuscitate" (DNR) / "Do not intubate" (DNI).

	Very Likely	Somewhat Likely	Unsure	Somewhat Unlikely	Very Unlikely	Prefer not to Answer
5. **How likely would you be to honor the advance directive form and not intubate the patient?**	◯	◯	◯	◯	◯	◯

<u>Please assign relative value to the items listed below as to the importance they played in your ultimate decision.</u>

	Very Important	Important	Neither Important or Unimportant	Unimportant	Very Unimportant	Prefer not to Answer
6. **Initial signing of advance directive is 5 years old.**	◯	◯	◯	◯	◯	◯
7. **Fear of legal liability if advance directive is honored or not honored.**	◯	◯	◯	◯	◯	◯

<u>SCENARIO 2:</u> A 65 year old patient with well controlled hypertension and who has signed an advance directive order stating a wish to "pass away in peace" arrives to the Emergency Department complaining of "chest pain". He is admitted to the cardiac telemetry unit pending a full work-up for cardiac ischemia. Shortly following arrival to the unit, he falls into ventricular fibrillation. A "Code" is called and upon your arrival to the bedside, the patient is apneic and requiring mask ventilation by the nursing staff.

	Very Likely	Somewhat Likely	Unsure	Somewhat Unlikely	Very Unlikely	Prefer not to Answer
8. **How likely would you be to honor the advance directive form and not electrically defibrillate the patient?**	◯	◯	◯	◯	◯	◯

<u>Please assign relative value to the items listed below as to the importance they played in your ultimate decision.</u>

	Very Important	Important	Neither Important or Unimportant	Unimportant	Very Unimportant	Prefer not to Answer
9. **Patient request to be allowed to "pass away in peace" does not mirror the acuity of the present condition.**	◯	◯	◯	◯	◯	◯
10. **Fear of legal liability if advance directive is honored or not honored.**	◯	◯	◯	◯	◯	◯

----------Please Complete Survey on Page 2------>

<u>SCENARIO 3</u>: A 68 year old patient with hypertension, diabetes, end stage renal disease on dialysis and acute lymphocytic leukemia arrives to the Emergency Department febrile, tachypneic and hypotensive. He is transferred to the intensive care unit (ICU) you are covering and upon arrival becomes asystolic. The hospital electronic charting system shows that the patient completed and signed an advance directive indicating "Do not resuscitate" (DNR) / "Do not intubate" (DNI) wishes during a recent visit with his oncologist. The wife of the patient has demanded that you "disregard the advance directive and do everything you can to save my husband".

	Very Likely	Somewhat Likely	Unsure	Somewhat Unlikely	Very Unlikely	Prefer not to Answer
11. How likely would you be to honor the advance directive form and not intubate the patient?	○	○	○	○	○	○

Please assign relative value to the items listed below as to the importance they played in your ultimate decision.

	Very Important	Important	Neither Important or Unimportant	Unimportant	Very Unimportant	Prefer not to Answer
12. Patient's wife demands that you disregard the patient's advance directive and resuscitate her husband.	○	○	○	○	○	○
13. Fear of legal liability if advance directive is honored or not honored.	○	○	○	○	○	○

UNRELATED TO THE PREVIOUS SCENARIOS, please indicate your level of agreement or disagreement with the following statements

	Strongly Agree	Agree	Neither Agree or Disagree	Disagree	Strongly Disagree	Prefer not to Answer
14. The legal liability involved in maintaining someone alive against their will (as directed by the wording of their advance directive) is less than the risk of mistakenly allowing them to die.	○	○	○	○	○	○
15. The term "comfort measures only" within the text of a patient's advance directive should also enable a physician to continue life support measures.	○	○	○	○	○	○
16. A patient's advance directive should be interpreted literally. "No life support" means no life support measures should be undertaken.	○	○	○	○	○	○
17. Because patients do not have the knowledge to adequately appreciate the idiosyncrasies involved in the practice of medicine, physicians should independently evaluate what is in the best interest of patients regardless of the contents of an advance directive.	○	○	○	○	○	○
18. A physician's decision to ignore a patient's advance directive should invoke legal liability only in the event of intentional disregard of patient wishes.	○	○	○	○	○	○
19. The financial cost of providing future medical care to a patient should never impact a physician's decision to either follow or disregard the advance directive.	○	○	○	○	○	○

----------------Thank you for your feedback---------------
